# Fatigue in stroke survivors: a 5-year follow-up of the Fall study of Gothenburg

**DOI:** 10.1007/s00415-023-11812-0

**Published:** 2023-06-15

**Authors:** Lior Schnitzer, Per-Olof Hansson, Carina M. Samuelsson, Avril Drummond, Carina U. Persson

**Affiliations:** 1 Department of Molecular and Clinical Medicine, Institute of Medicine, Sahlgrenska Academy, University of Gothenburg, Gothenburg, Sweden; 2 Department of Medicine, Geriatrics and Emergency Medicine, Sahlgrenska University Hospital/Östra, Region Västra Götaland, Gothenburg, Sweden; 3 Department of Occupational Therapy and Physiotherapy, Sahlgrenska University Hospital/Östra, Region Västra Götaland, Gothenburg, Sweden; 4 Faculty of Medicine and Health Sciences, University of Nottingham, Nottingham, UK; 5 Department of Clinical Neuroscience, Rehabilitation Medicine, Institute of Neuroscience and Physiology, University of Gothenburg, Gothenburg, Sweden

**Keywords:** Stroke, Post-stroke fatigue, Observational study, Fatigue Assessment Scale

## Abstract

Longer term knowledge of post-stroke fatigue (PSF) is limited. Our aim was to describe the prevalence of, and to identify baseline predictors associated with, PSF 5 years after stroke. We undertook a follow-up of stroke survivors from the 504 consecutively recruited participants in the observational “The Fall Study of Gothenburg”, conducted between 2014 and 2016. The dependent variable, PSF, was assessed using the Swedish version of the Fatigue Assessment Scale (S-FAS) and defined as having a S-FAS score ≥ 24. The S-FAS questionnaire was mailed to potential participants in August 2020. The independent variables, previously obtained from medical records, included age; sex; comorbidities; stroke severity; hospital length of stay; body mass index (BMI); number of medications and lifestyle factors at index stroke. To identify predictors of PSF, univariable and multivariable logistic regression analyses were performed. Of the 305 eligible participants, 119 (39%) responded with complete S-FAS. Mean age at index stroke was 71 (SD 10.4) years and 41% were female. After a mean of 4.9 years after stroke, the prevalence of PSF was 52%. Among those with PSF, almost two thirds were classified as having both physical and mental PSF. In the multivariable analysis, only high BMI predicted PSF with an odds ratio of 1.25 (95% CI 1.11–1.41, p < 0.01). In conclusion, half of the participants reported PSF 5 years after index stroke and higher body mass index was identified as a predictor. The findings from this study are important for healthcare professionals, for planning health-related efforts and rehabilitation of stroke survivors.

*ClinicalTrials.gov, Identifier* NCT02264470.

## Introduction

Stroke is a leading cause of morbidity and mortality worldwide, with 6.5 million deaths and 143 million disability-adjusted life years (DALYs) reported in 2019 alone [1]. A common yet often overlooked sequela of stroke, and an indicator of poor functional outcome, is post-stroke fatigue (PSF) [2–4]. PSF has been defined as pathological fatigue of chronic nature which is not alleviated by rest [5]. Reported prevalence of PSF varies considerably, from 39 to 72%, in both the immediate-term, and up to 2 years after stroke [2, 6–10]. There are few studies on long-term prevalence of PSF, here defined as five or more years after index stroke [11–15]. Studies have reported a prevalence ranging between 37% [12] and 80% [13], similar to prevalence figures found shortly after stroke [2, 6–10]. The severity of PSF is also prone to considerable variation [16]. In milder cases, PSF has been described as “a manageable fatigue” [16]. However, in more severe cases, PSF is a debilitating condition with substantial implications for quality of life and participation in rehabilitation [5]. PSF has recently been identified as one of ten top research-priorities relating to life after stroke, in view of the high prevalence and day-to-day implications of it [17].

Gaining more insight into the predictors of this complex clinical entity is crucial, as this knowledge may facilitate the identification of at-risk patients from the outset of rehabilitation. If the predictors are modifiable, this could be useful for assisting patients towards a significant recovery. Previous observational studies have identified the following variables as potential predictors of PSF, mainly studied within 12 months after stroke [4, 6, 7, 18–20]: medical comorbidities (hypertension, diabetes mellitus, arrythmias), female sex [4, 13, 20, 21] at 3 months to 7 years after stroke, marital status [6, 18] at 6 months or less after stroke, age at 1 [19] to 2 years [2] after stroke, higher body mass index (BMI) [22] at 2 years after stroke, smoking [23] at 15 months after stroke, number of medications[7] at 6 months after stroke. Stroke severity, expressed as a higher score on the National Institutes of Health Stroke Scale (NIHSS) has been shown to be a predictor of PSF at 6 [12] to 7 [13] years after stroke. However, research on early predictors of PSF at 5 years or longer after stroke is still limited [12, 13, 15]. Therefore, the aim was to describe the prevalence of, and to identify baseline predictors associated with, PSF 5 years after stroke. Based on clinical expertise and previous research, our hypotheses were that PSF is common and affects the majority of stroke survivors [13, 14], and that increased number of medications [18], older age, higher BMI [22], as well as the presence of diabetes mellitus [21], hypertension [21], ischemic heart disease [24], atrial fibrillation, congestive heart failure, and physical inactivity prior to index stroke [25] are associated with higher prevalence of PSF 5 years after stroke onset.

## Methods

### Study design

This study used a cross-sectional and longitudinal design to conduct a 5-year follow-up of the observational cohort study, the Fall Study of Gothenburg (FallsGOT) [26].

#### Inclusion and exclusion criteria

The FallsGOT had a consecutive recruitment of patients with a clinical diagnosis of stroke, admitted to the stroke unit at the Sahlgrenska University Hospital, Östra in Gothenburg, Sweden between 1st October 2014 and 30th June 2016. In the current study, which we are reporting here, we followed up those patients who were still alive and living in the Västra Götaland region in August 2020. Further details of the FallsGOT are described elsewhere [26–34]. The Swedish Ethical Review Authority approved this present follow-up study on May 20th, 2020, (Reference numbers: 2019-06476, 2020-05577).

#### Assessment of the independent variables at index stroke

The following independent variables were collected at index stroke, and were available from the FallsGOT database: age; sex; BMI; stroke severity (assessed using NIHSS [35]); length of hospital stay; number of medications; smoking status; presence of comorbidities (more specifically: hypertension; diabetes mellitus; atrial fibrillation; congestive heart failure and ischemic heart disease) and self-reported level of physical activity level prior to the index stroke using the Saltin–Grimby Physical Activity Level Scale (SGPALS) [36]. The SGPALS is a well-established four-level scale, where a higher score indicates higher physical activity level [36].

#### Assessment of the primary outcome, PSF

The dependent variable, PSF, was assessed in August 2020, when all the surviving 305 FallsGOT participants were sent a questionnaire pack, which included the Swedish Fatigue Assessment Scale (S-FAS) [37]. The individuals who did not reply were sent a reminder between September 16th and 29th 2020. The date on which the response letter was received was use as the follow-up date in our analysis. The S-FAS consists of 10 statements with five response categories each (1 = never, 2 = sometimes, 3 = regularly, 4 = often and 5 = always). The S-FAS assesses both physical and mental fatigue, where the statements 1, 2, 4, 5 and 10 assess physical fatigue, while the statements 3 and 6–9 assess mental fatigue [38]. The scores on statements 4 and 10 were recorded in reverse order (i.e., 1 = 5, 2 = 4, 3 = 3, 4 = 2, 5 = 1) according to the manual. The possible total score of fatigue ranges from 10 to 50, where a higher score indicates increased fatigue. Based on previous research [39], a cut-off score of S-FAS ≥ 24 was used to define the presence of PSF.

### Statistical analyses

The statistical analyses were conducted using the IBM Statistical Package for Social Sciences (SPSS) software, version 28. Descriptive statistics were presented by means and standard deviations for interval data, as medians and interquartile ranges (IQRs) for interval and ordinal data, and as numbers and percentages for nominal data. To identify predictors of PSF, univariable and multivariable regression analyses were performed. To be included in the multivariable logistic regression analysis, a level of significance at p ≤ 0.1 at the univariable analyses was used as a cut-off, except for the variables age and sex which were to be included as potential confounders in the multivariable analysis regardless of significance. Spearman’s rank correlation coefficient was used to exclude strong correlation between the independent variables. Correlation coefficients of ≥ 0.7 were considered multicollinear [40]. Results from the univariable and multivariable logistic regression analyses are presented as odds ratios (ORs) with 95% confidence intervals (CIs). For the multivariable analysis, the level of significance was set to p < 0.05 (two-tailed). The goodness-of-fit for the multivariable model was tested by using the Hosmer–Lemeshow test, the Cox & Snell and the Nagelkerke pseudo R^2^ statistics. The diagnostic ability of the multivariable analysis was investigated using the Receiver Operating Characteristic (ROC) curve, where 0.7–0.79 was considered acceptable, 0.8–0.89 was considered excellent and ≥ 0.9 was considered outstanding diagnostic accuracy [41].

#### Interval estimation

For incomplete S-FAS questionnaires, i.e., missing data regarding the dependent variable, interval estimation was used. Unanswered questions were completed with any hypothetical value (1–5), and only scores that after that remained on the same side of the cut-off, i.e., a S-FAS score < 24 or a S-FAS score ≥ 24, were included.

## Results

**Fig. 1:**
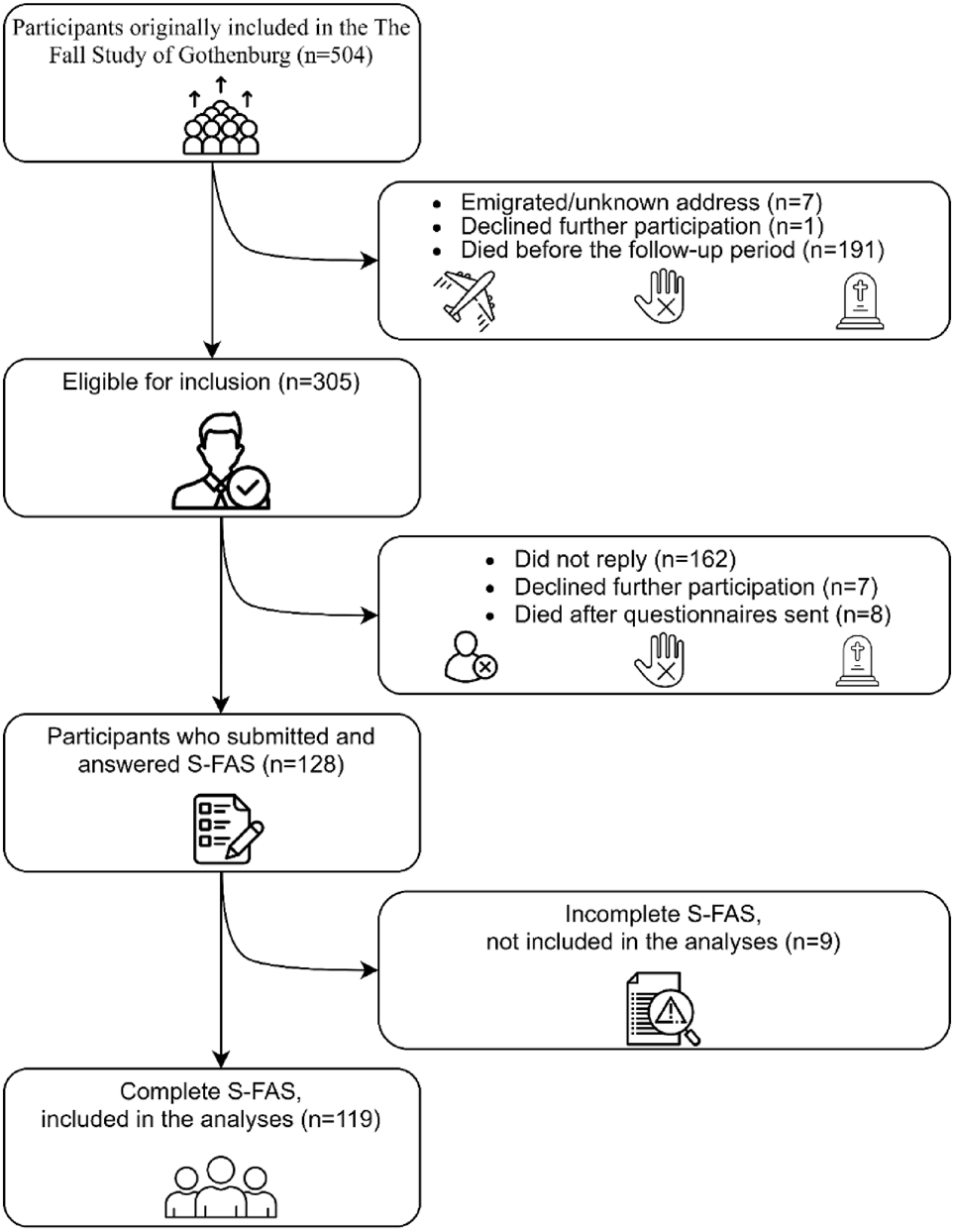
The flow chart for the study enrollment

The inclusion process is displayed in Fig. 1. The response rate was 42% (128 of 305 eligible participants). A total of nine S-FAS were incompletely answered. Although interval estimation was conducted, none of the incomplete S-FAS could be included. Consequently, the analyses were based on 119 participants. Table 1 presents the characteristics of the respondents, displayed by presence of fatigue, and the non-respondents, at index stroke. There was a slightly higher proportion of males and the population had predominantly had a mild ischemic stroke. All but one respondent had at least one comorbidity. In addition, at index stroke, around two thirds reported that they prior to the stroke had been physically active to some degree, and it was noted that more than four out of five were non-smokers. At a mean follow-up time of 4.9 (SD 0.5) years, 52% of the participants were classified as having PSF.

**Table 1:** Characteristics of the respondents, split by presence of fatigue, and non-respondents, at index stroke

Characteristic	All respondents (N = 128)	Respondents with a complete S-FAS (N = 119)		Non-respondents (N = 177)
		No fatigue (S-FAS < 24) (N = 57)	Fatigue (S-FAS ≥ 24) (N = 62)	
Age, years, mean (SD)	70.7 (10.4)	69.8 (9.4)	71.2 (11.2)	73.1 (12.0)
Sex, n (%)				
Female	57 (44.5)	26 (45.6)	23 (37.1)	85 (48)
Male	71 (55.5)	31 (54.4)	39 (62.9)	92 (52)
Duration of follow-up, years, mean (SD)	4.9 (0.5)	4.9 (0.5)	5.0 (0.52)	–
Type of stroke, n (%)				
Infarction	122 (95.3)	56 (98.2)	57 (91.9)	165 (93.2)
Hemorrhage	6 (4.7)	1 (1.8)	5 (8.1)	12 (6.8)
OSCP classification, n (%)				
TACI	3 (2.5)	1 (1.8)	1 (1.8)	3 (1.8)
PACI	38 (31.1)	14 (25.0)	19 (33.3)	50 (30.3)
POCI	45 (36.9)	28 (50.0)	16 (28.1)	48 (29.1)
LACI	36 (29.5)	13 (23.2)	21 (36.8)	64 (38.8)
Stroke localization, n (%)				
Right side lesion	60 (46.9)	20 (35.1)	34 (54.8)	103 (58.2)
Left side lesion	61 (47.7)	32 (56.1)	26 (41.9)	68 (38.4)
Bilateral lesion	3 (2.3)	2 (3.5)	1 (1.6)	3 (1.7)
Unknown location	4 (3,1)	3 (5.3)	1 (1.6)	3 (1.7)
NIHSS score, median (IQR)	1 (0–2)	0 (0–1)	1 (0–3)	2 (0–3)
Length of stay, days, mean (SD)	7.9 (6.3)	6.6 (4.7)	8.6 (7)	11.7 (11.3)
Number of medications, mean (SD)	5.5 (2.7)	4.9 (2.5)	6.1 (2.8)	6.7 (3.5)
Comorbidity, n (%)				
Hypertension	94 (73.4)	40 (70.2)	46 (74.2)	136 (76.8)
Diabetes mellitus	24 (18.8)	11 (19.3)	11 (17.7)	46 (26)
Atrial fibrillation	25 (19.5)	13 (22.8)	10 (16.1)	38 (21.5)
Congestive heart failure	10 (7.8)	5 (8.8)	4 (6.5)	12 (6.8)
Ischemic heart disease	24 (18.8)	11 (19.3)	12 (19.4)	39 (22)
BMI, kg/m^2^, mean (SD)	26.9 (4.7)	25.1 (3.6)	28.7 (5.1)	26.7 (4.98)
BMI, kg/m^2^, median (IQR)	26 (23.7–29.1)	24.5 (23–26.9)	28.3 (24.5–31.6)	26.5 (23.9–29.3)
Previous physical activity level, n (%)				
Sedentary	43 (33.6)	13 (22.8)	25 (40.3)	103 (58.9)
Physically active	85 (66.4)	44 (77.2)	37 (59.7)	72 (41.1)
Smoking, n (%)				
No	70 (57.9)	32 (60.4)	34 (55.7)	84 (49.7)
Previous smoker	30 (24.8)	15 (28.3)	14 (23)	40 (23.7)
Current smoker	21 (14.4)	6 (11.3)	13 (21.3)	43 (25.4)

Previous physical activity level was assessed using the Saltin–Grimby Physical Activity Scale (SGPALS). Here sedentary refers to the participants who scored 1, and physically active refers to the participants who scored 2, 3 or 4, on the SGPALS. Previous smoker refers to participants who have ceased smoking at least 1 month previously

*S-FAS* the Swedish Fatigue Assessment Scale, *SD* standard deviation, *IQR* interquartile range, *OCSP* Oxfordshire Community Stroke project, *TACI* total anterior circulation infarction, *PACI* partial anterior circulation infarction, *POCI* posterior circulation infarction, *LACI* lacunar infarction, *NIHSS* the National Institutes of Health Stroke Scale, *BMI* body mass index

Table 2 presents the ORs for the early prediction of PSF during the follow-up based on the univariable and multivariable analyses. In the univariable analysis, stroke severity (expressed as a higher NIHSS score), higher number of medications, higher BMI at index stroke, and self-reported physical inactivity prior the stroke, were found to be statistically significant (p ≤ 0.05). In the multivariable analysis, higher BMI at index stroke was the only variable that remained as a statistically significant predictor of PSF 5 years after stroke.

**Table 2:** Odds ratios (ORs) for prediction of post-stroke fatigue 5 years after stroke based on univariable and multivariable analyses

Potential predictors	*	Data presen-tation	Univariable analysis		Multivariable analysis	
			OR (95% CI)	*P*	OR (95% CI)	*P*
Age	0	Years	1.01 (0.98–1.05)	0.47	1.01 (0.96–1.06)	0.76
Sex	0	Male (Ref.)	1			
		Female	0.7 (0.34–1.46)	0.35	0.67 (0.26–1.73)	0.41
NIHSS	7	Points	1.39 (1.07–1.79)	0.01	1.22 (0.95–1.56)	0.12
Length of stay	0	Days	1.07 (0.99–1.15)	0.07	1.08 (0.98–1.2)	0.09
Number of medications	1	Number	1.21 (1.04–1.41)	0.01	1.14 (0.94–1.37)	0.18
Hypertension	0	No (Ref.)	1			
		Yes	1.22 (0.55–2.73)	0.63		
Diabetes Mellitus	0	No (Ref.)	1			
		Yes	0.9 (0.36–2.28)	0.83		
Atrial fibrillation	0	No (Ref.)	1			
		Yes	0.65 (0.26–1.63)	0.36		
Congestive heart failure	0	No (Ref.)	1			
		Yes	0.72 (0.18–2.81)	0.63		
Ischemic heart disease	0	No (Ref.)	1			
		Yes	1.00 (0.4–2.5)	0.99		
BMI	1	Kg/m^2^	1.23 (1.11–1.36)	< 0.001	1.26 (1.11–1.43)	< 0.001
Self-reported physical activity level	0	Physically Active (Ref.)	1			
		Physically inactive	2.29 (1.03–5.09)	0.04	1.05 (0.37–3.01)	0.93
Smoking	5	No (Ref.)	1			
		Previous	0.88 (0.37–2.11)	0.77		
		Current	2.04 (0.69–6.01)	0.20		

In the univariable analyses, the criterion for significance was p ≤ 0.1. Included in the multivariable analysis (n = 111). The Hosmer–Lemeshow test; p 0.03; the Cox/Snell R^2^ 0.28; the Nagelkerke R^2^ 0.37. Area under the receiver operating characteristic curve 0.82 (95% CI 0.74–0.9, p < 0.001)

*P*: p-value; CI: confidence interval; NIHSS: the National Institutes of Health Stroke Scale; BMI: body mass index

*Missing data

**Fig. 2:**
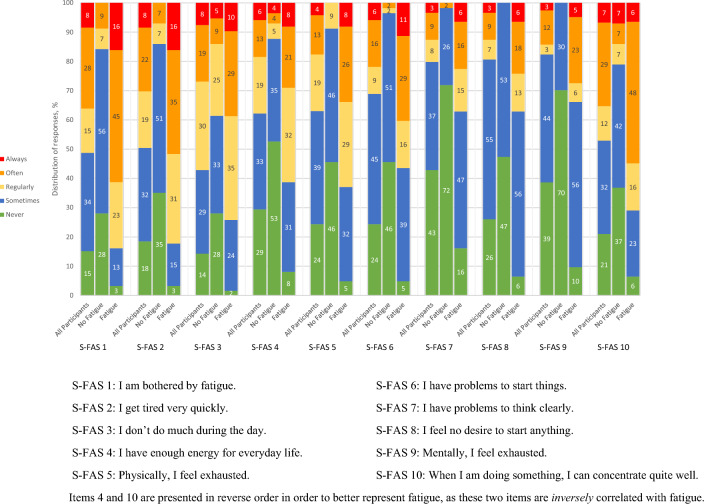
The participants’ response patterns for the Swedish Fatigue Assessment Scale (S-FAS) 5 years after a stroke by all participants/respondents with a complete S-FAS, by those classified as not having fatigue (n = 57) and those classified as having fatigue (n = 62)

The participants’ response patterns for the S-FAS for each of the ten statements by all the respondents and by the respondents with and without fatigue can be found in Fig. 2. Fewer than one tenth of all respondents stated that they ‘always’ experienced fatigue. Of the respondents with PSF, more than half stated that they were ‘often’ or ‘always’ bothered by fatigue and got tired very quickly (items 1 and 2). About one third had ‘never’ or ‘sometimes’ enough energy for everyday life (item 4). Four out of ten had problems initiating things (item 6). A quarter of the participants with fatigue stated that they had problems in thinking clearly and felt mentally exhausted (items 7 and 9). More than half stated that they ‘never’ or ‘sometimes’ concentrated well when doing something (item 10). Among participants with PSF, twice as many reported *having problems* initiating things than having *no desire* to start things often or always. Among those with PSF, almost two thirds were classified as having both physical and mental PSF. Almost one third (32%) was classified as having physical PSF solely and three participants (5%) had mental PSF solely, defined as a score of ≥ 12 points in the statements relating to either physical (items 1, 2, 4, 5, 10) or mental (items 3, 6–9) PSF.

Among those without PSF, generally at least four in five participants never or sometimes experienced any form of fatigue, i.e., answered ‘never’ or ‘sometimes’ to all statements except for “I don’t do much during the day”.

## Discussion

In this study we aimed to describe the prevalence of, and to identify baseline predictors associated with, PSF at follow-up, approximately five years after stroke. We found that more than half of the participants reported PSF (defined as S-FAS score ≥ 24). Our hypothesis, that higher BMI was a predictor of PSF, was confirmed. However, our remaining hypotheses were rejected, i.e., older age, greater stroke severity, increased number of medications, as well as the presence of diabetes mellitus, hypertension, ischemic heart disease, atrial fibrillation and congestive heart failure at index stroke and physical inactivity prior to the stroke, were not identified as predictors of PSF in the current study. However, and of note, to our knowledge, no other study has investigated the association between multiple potential predictors in a post-stroke population where both hemorrhagic and ischemic stroke are included for such a long follow-up period.

The prevalence of PSF reported here is within the previously reported range [12, 13]. Nevertheless, previous studies have shown conflicting results regarding the association between PSF and stroke severity several years after stroke, where an association between stroke severity and PSF was found in two studies [12, 13], but not in another [18]. Moreover, our current study shows that even individuals with a mild stroke can have PSF. Given the incidence of stroke and the distribution of responses to the S-FAS, this implies a potentially high burden of fatigue for both patients and society.

Our findings contrast with previous studies that have identified age, sex [13], greater stroke severity [13], hypertension [18, 21], diabetes mellitus [21], atrial fibrillation [21], physical inactivity and smoking [23] as potential predictors of PSF. However, this could be explained by the fact that these studies had significantly shorter follow-up periods of up to 15 months [4, 23] or were based on younger populations [12, 13]. Other possible explanations are that the population in our study was too small or non-representative.

The results from the current study provide support to the idea of an association between obesity, defined as BMI greater or equal to 30 kg/m^2^, and fatigue, found by Gu et al. [22] 2 years after stroke. An association between PSF and obesity may have far-reaching implications as obesity becomes increasingly common [42]. Obesity is also independently associated with fatigue in adult individuals [43] as well as a known modifiable risk factor for stroke [44], and therefore an association between obesity and PSF is likely. In addition, there is a well-known link between PSF and obstructive sleep apnea (OSA), which is common in people with obesity [45]. Therefore, a higher prevalence of OSA among obese people may, to some extent, explain the association between obesity and fatigue. The efforts to curtail this modifiable risk factor may best be achieved by health-promotion at population level.

This study has some limitations including the relatively low response rate. This, however, was expected and could be explained by the fact that the 504 FallsGOT participants were at a mean age of 77 years at baseline [26] and an additional 5 years older at follow-up with the risk of having suffered additional comorbidity, potentially affecting the ability or willingness to participate in the current follow-up. Another limitation is that the generalizability of our results is based on data from younger survivors of predominately mild strokes, conservatively treated (as none of the study participants were initially treated with thrombolysis or thrombectomy). Thus, we need to be careful of extrapolating this finding more widely. However, as most people have a mild stroke, both in Sweden and globally [46–48], our findings are likely to be generalizable to a significant proportion of stroke survivors. The current study design does not provide information about a potential causal relationship between BMI and PSF. Furthermore, over the course of 5 years in an aging population, more/other diagnoses may have been added, which might have had an impact on how the participants responded to the statements in the S-FAS. Nevertheless, the major strength of this study is the long follow-up period, in addition to the consecutive inclusion at index stroke and the use of the S-FAS, which is a validated and reliable scale for assessing PSF [37]. Moreover, in our study we investigated several different potential predictors relating to participants’ health conditions, body functions and structures, as well as personal factors.

Survival after stroke has increased in recent years and for all stroke survivors, quality of life after stroke is important. As previously noted, PSF is associated with a significant decrease in quality of life. Thus, reducing the burden of PSF is a challenge that needs to be undertaken and this is reflected in the fact that PSF is one of the International Stroke Recovery and Rehabilitation Alliance (SSRA) round table topics, English et al. Post stroke fatigue: A position paper (Paper in preparation). Further investigation of the pathophysiology behind PSF is also mandated, especially if it can reveal any causal relationship between being overweight, obesity and having PSF. Further research on PSF using optimal and individualized lifestyle interventions (so-called precision health) in overweight stroke survivors should be pursued.

In conclusion, we found that half of the respondents reported PSF approximately 5 years after index stroke and higher body mass index was identified to be a predictor. These findings are important for healthcare professionals, for planning health-related efforts such population-based primary prevention, and for implementing regular follow-ups to support those in need of rehabilitation.

## Data Availability

The data that support the findings of this study are available from the corresponding author, [CUP], upon reasonable request. According to Swedish regulations, permission to use data can be obtained after an application to and approval by the ethics committee.
